# Some binocular advantages for planning reach, but not grasp, components of prehension

**DOI:** 10.1007/s00221-019-05503-4

**Published:** 2019-03-08

**Authors:** Simon Grant, Miriam L. Conway

**Affiliations:** Applied Vision Research Centre, City, University of London, Northampton Square, London, EC1V 0HB UK

**Keywords:** Visuomotor behaviour, Stereopsis, Monocular vision, Internal model, Visual feedback, Online control

## Abstract

Proficient (fast, accurate, precise) hand actions for reaching-to-grasp 3D objects are known to benefit significantly from the use of binocular vision compared to one eye alone. We examined whether these binocular advantages derive from increased reliability in encoding the goal object’s properties for feedforward planning of prehension movements or from enhanced feedback mediating their online control. Adult participants reached for, precision grasped and lifted cylindrical table-top objects (two sizes, 2 distances) using binocular vision or only their dominant/sighting eye or their non-dominant eye to program and fully execute their movements or using each of the three viewing conditions only to plan their reach-to-grasp during a 1 s preview, with vision occluded just before movement onset. Various kinematic measures of reaching and grasping proficiency, including corrective error rates, were quantified and compared by view, feedback and object type. Some significant benefits of binocular over monocular vision when they were just available for pre-movement planning were retained for the reach regardless of target distance, including higher peak velocities, straighter paths and shorter low velocity approach times, although these latter were contaminated by more velocity corrections and by poorer coordination with object contact. By contrast, virtually all binocular advantages for grasping, including improvements in peak grip aperture scaling, the accuracy and precision of digit placements at object contact and shorter grip application times preceding the lift, were eliminated with no feedback available, outcomes that were influenced by the object’s size. We argue that vergence cues can improve the reliability of binocular internal representations of object distance for the feedforward programming of hand transport, whereas the major benefits of binocular vision for enhancing grasping performance derive exclusively from its continuous presence online.

## Introduction

Reaching-to-grasp an object involves a complex sequence of target-encoding, decision-making and control mediated by predictive/feedforward programming combined with reactive/feedback mechanisms. There is continuing debate over the extent to which such goal-directed hand movements are programmed in advance or controlled online (Jeannerod 1984; Desmurget and Grafton 2000; Elliot et al. 2010, 2017; Wolpert et al. 2011; Gaveau et al. 2014; Zhao and Warren 2015), but no dispute that vision makes critical contributions to both processes. In this context, there is substantial evidence that binocular vision usually provides significant benefits over monocular viewing for efficient (fast, accurate and precise) prehension performance in both ‘real’ (Servos et al. 1992; Servos and Goodale 1994; Jackson et al. 1997; Watt and Bradshaw 2000; Loftus et al. 2004; Melmoth and Grant 2006; Gnanaseelan et al. 2014) and ‘virtual’ (Bingham et al. 2001; Greenwald et al. 2005; Knill 2005) 3D environments. The present study examines whether these normal binocular advantages are associated with the planning or just the online control of all or only some sub-components of ‘natural’ reach-to-grasp movements.

Programming the motor commands for prehension typically begins with visually encoding the target’s properties. Classically (Jeannerod 1984), evaluation of its distance is used to plan the appropriate reach trajectory, velocity and amplitude, with assessments of its 3D size/shape (i.e., solidity) a key element in deciding the optimal grip configuration. Evidence from selective perturbation studies suggests that binocular viewing can provide exclusive sources of each type of information to normal subjects—from ocular vergence and horizontal retinal disparity, respectively (Mon-Williams and Dijkerman 1999; Bradshaw et al. 2004; Melmoth et al. 2007, 2009; Niechwiej-Szwedo et al. 2012). These binocular cues, when operating in isolation, are prone to distortion and/or contraction biases in which the distance and depth of near objects are overestimated and underestimated for those further away (Foley 1980; Johnston 1991; Brenner and Van Damme 1999; Tresillian et al. 1999; Hibbard and Bradshaw 2003; Volcic et al. 2013), but when integrated with complementary sources of monocular information available in each eye appear to enhance the reliability of evidence gathered during the planning process (Knill 2005; Keefe et al. 2011). According to accounts that emphasize the pre-eminence of internal models for producing desired motor outputs, binocular viewing should thus provide a more dependable basis for predictive control of prehension than when the goal object and its surroundings can be seen with only one eye. Consistent with this position, it has been repeatedly shown that normal subjects make many fewer obvious errors or corrections during the later stages of their reach and grasp under binocular compared to monocular viewing conditions (e.g., Marotta and Goodale 1998, 2001; Bradshaw et al. 2004; Melmoth and Grant 2006; Melmoth et al. 2009), implying that differences between binocular prehension programming and the executed movement may be negligible.

On the other hand, there is contradictory evidence that human observers are generally poor are judging object solidity (Lee et al. 2008) and can exhibit the same systematic distortions in distance estimation and depth constancy for both perception and action control, even when they are able to combine multiple task-relevant binocular and monocular cues to these object properties (Tittle et al. 1995; Bingham et al. 2000; Todd and Norman 2003; Bozzacchi and Domini 2015; Kopiske et al. 2019). This would further imply that any differences in the efficacy of binocular versus monocular vision for prehension planning may be negligible, since the visuomotor system is only capable of constructing ‘weak’ and unreliable internal representations of intended movements, even under optimal data-gathering conditions. In fact, there is substantial evidence that the main and constant advantage of binocular vision is in providing feedback about changes in the position of the moving hand and digits relative to the target for online control of the reach and grasp (Servos et al. 1992; Jackson et al. 1997; Bradshaw and Elliot 2003; Loftus et al. 2004; Melmoth and Grant 2006; Anderson and Bingham 2010). These findings support an alternative interpretation for the relative absence of errors and abrupt corrections during binocular reach-to-grasps: that continuous regulation, mediated by dynamic disparity processing, results in fast and subtle online adjustments to the movements. Indeed, the binocular advantages for prehension, including lower overt error- and correction-rates, are lost under conditions of binocular feedback in which such hand–target disparities are not available to be exploited online or are no more reliable than alternative cues provided by monocular vision (Bradshaw et al. 2004; Loftus et al. 2004; Greenwald and Knill 2009; Keefe and Watt 2017).

Several studies cited above that have undertaken detailed kinematic analyses of binocular compared to monocular performance, however, have argued that reach-to-grasp execution involves a sequence of sub-movements which are under differential mechanisms of feedforward and/or feedback control (Watt and Bradshaw 2000; Bradshaw and Elliot 2003; Loftus et al. 2004; Melmoth and Grant 2006). The first component of these multiple processes comprises parameters of the initial reach—such as its peak velocity (PV)—up to and including its peak deceleration, which seem to be mainly products of feedforward programming and for which binocular vision is reported to provide no (Servos and Goodale 1994; Watt and Bradshaw 2000; Bradshaw and Elliot 2003) or only minor/inconsistent (Bradshaw et al. 2004; Loftus et al. 2004; Melmoth and Grant 2006) advantages over one eye alone. The next is the main hand–target feedback stage outlined above, which encompasses the low velocity end-phase of the reach and is mainly concerned with preparing the initial grasp, via formation of the peak grip aperture (PGA), and then closing it upon the goal object. The PGA is an accepted measure of the target’s inferred 3D shape and size established when planning the grasp (Jeannerod 1984; Melmoth and Grant 2006) and is usually well scaled to the goal object’s dimensions when binocular disparity and monocular cues can be integrated in just the pre-movement period (Jakobson and Goodale 1991; Keefe and Watt 2009; Keefe et al. 2011). Last is the post-contact phase during which the grip is applied and the object lifted. These sub-actions, too, derive major benefits from binocular vision during the preceding hand–target stage, since monocular viewing in this period is associated with inaccurate and imprecise thumb and finger contacts with the goal object and with poor coordination between these contacts and reach termination (Servos and Goodale 1994; Melmoth and Grant 2006; Melmoth et al. 2009), necessitating more reliance on haptics for achieving grip stability and causing delays in load and/or lift force application. In sum, these analyses lead us to hypothesise that binocular vision normally provides some advantage for programming key aspects of the grasp, but not the reach, with major benefits for feedback control in the hand–target approach which should be lost in the absence of online disparity information.

A fairly simple method for testing this hypothesis is to compare various kinematic measures of prehension performance when vision is available both for programming the movements and for their subsequent online control with open-loop conditions in which it is available only for planning. This we have done. More specifically, we compared binocular with monocular (dominant eye and non-dominant eye only) reach-to-grasps performed under full vision (FV) or no visual feedback (NVF) conditions. Our logic was that any binocular advantage over monocular viewing present in the FV condition that persist in the absence of visual feedback derive from the internal spatial representation of the task generated at the planning stage, while those that disappear with NVF available are products of online control.

Binocular closed- versus open-loop prehension has been examined before. Typical findings are that people adopt a cautious strategy in the absence of visual feedback (Jakobson and Goodale 1991; Churchill et al. 2000; Loftus et al. 2004; Watt and Bradshaw 2003; Whitwell et al. 2008), by slowing down and prolonging their movements, particularly in the hand–target phase, and/or by producing relatively earlier and wider PGAs. These safety-first measures appear designed to ensure that the hand does not collide heavily with or completely miss the intended target, and are incompatible with highly reliable feedforward control, including of the PGA, especially as thumb and finger placements at object contact exhibit inaccuracies and imprecisions under both binocular (Churchill et al. 2000; Melmoth and Grant 2012) and monocular (Westwood et al. 2005) NVF conditions, analogous to increases in constant and variable errors routinely observed in open-loop single digit pointing experiments (Elliot et al. 2010). But only Jackson et al. (1997) have directly compared binocular with monocular closed- versus open-loop prehension as in the present study, and they found—contrary to our hypothesis—that only one aspect of reach programming (faster PVs) retained some binocular advantage in the absence of online vision.

A further complexity, however, is that the normal benefits of binocular vision can vary systematically with spatial target properties linked to the accuracy demands of the task (see Servos et al. 1992; Jackson et al. 1997; Bradshaw et al. 2004; Melmoth and Grant 2006; Keefe et al. 2011). For example, we found that those for both early (PV) and later (LVP, correction-rate) reach parameters were most marked for targets located around arm’s length rather than closer to the body, when the increased amplitude and duration of hand transport provides more opportunity for corrupting effects of noise to accumulate in the visuomotor system (Harris and Wolpert 1998). We also found that binocular advantages for accurate scaling of the PGA and grasp size at object contact were most evident for a small compared to larger object, which had a relatively restricted grip contact surface and was easy to topple over. We thus hypothesized further that any loss of advantage when binocular vision was only available at the planning stage would likely be most evident for reaches to ‘far’ targets and when grasping a ‘smaller’ object.

For these various reasons, we examined multiple dependent measures reflecting the planning or online control (including error/correction-rates) of the reach and grasp produced under the three views to cylindrical objects of 2 (small/large) diameters presented at 2 (near/far) distances on blocked FV followed by NVF trials. We ran trials randomly interleaved by view to minimize stereotypical behaviour and blocked rather than randomized or interleaved by feedback because subjects are reported to make strategic changes to their normal FV behaviour under these latter conditions, either as a precaution that feedback might actually be removed during the movement (Jakobson and Goodale 1991) or on the basis of the trial’s feedback history (Whitwell et al. 2008). We chose to always present the FV before the NVF blocks, rather than counter-balancing their order between subjects, to ensure that any binocular advantages retained with NVF available could not be due to unfamiliarity with the general task/experimental conditions, which would likely have more deleterious effects on monocular performance (Marotta and Goodale 2001; Keefe and Watt 2009).

## Materials and methods

### Subjects

Participants were 20 adults (8 males) aged 19–36 years (median = 22) with normal or corrected-to-normal vision (via contact lens wear), and high-grade stereo-acuity of at least 40 arc secs (Wirt-Titmus test, Stereo Optical Co. Inc., Chicago, USA). Most (*n* = 13) were strongly right-handed and 7 were left-handed as determined via self-report on the abbreviated Edinburgh Handedness Inventory questionnaire (Oldfield 1971). Procedures were approved by the Senate Ethical Committee of City, University of London and were conducted according to 1964 Helsinki Declaration standards.

### Hand movement recordings

Subjects sat at a black table (60 cm wide × 70 cm deep) gripping a circular button (3 cm diameter) positioned 12 cm along their midline, between the thumb and index finger of their preferred hand. The button operated as the fixed start and end hand location for each movement trial. Lightweight infrared reflective markers (7 mm diameter) were placed on the wrist (head of radius) and on the opposing thumb and finger nails of this hand. The instantaneous positions of the three markers in 3D space, and of another marker fixed to the centre of the upper surface of the target object, were tracked at a sampling rate of 60 Hz and spatial resolution of < 0.4 mm by three infrared motion-capture cameras (ProReflex, Qualisys AB, Sweden) triangulating the table from above. Target objects were cylindrical white (i.e., high-contrast) dowels of the same height (100 mm) but of either small (23 mm, 32 g) or large (46 mm, 128 g) diameter, placed at either a near (25 cm from the start button along the midline) or far location (40 cm from the start button at 10 deg from the midline on the side of the preferred hand) on different trials. These selections were based on their use in previous experiments. For example, we know that the two object sizes are amenable to precision grasping across a range of subject hand sizes, with their different (midline versus off-midline) target locations requiring different initial reach directions and digit trajectories across trials, adding some variety to the relatively simple tasks. Participants wore PLATO liquid crystal goggles (Translucent Technologies Inc., Toronto, Canada). These were opaque in the resting state, but made independently transparent to generate binocular, monocular dominant (sighting) eye or non-dominant eye views at the start of different trials.

In the first part of the experiment, subjects completed 2 separate blocks of 24 trials each comprising pseudo-randomized sequences (identical trial-types were not presented consecutively) of the 3 views by 2 object sizes by 2 distances combination repeated twice. These initial trials were conducted under FV conditions, in which participants could see the target when planning their movement and both the object and their moving hand during its execution. More specifically, opening of both or only one of the goggle lenses was the cue for the subjects to begin their reach, with the lenses closing 5 s later, by which time they had picked up the object and returned their hand to the start position. In the second part, participants repeated the two trial blocks, but under NVF conditions, in which they could see the goal-object only during the planning stage. On these trials, the goggles opened for 1 s to allow a binocular or monocular ‘preview’ of the target, with sudden return of the lenses to the opaque state being the cue to move. For both conditions, subjects were told to move as ‘naturally and accurately as possible’—with speed not being of the essence—and to use a precision grip to pick up the object with the thumb and finger aimed at about half its height, before placing to it on the table on the same side as their moving hand and returning to the start button. A few practice trials under each view were provided before each part of the test, until the experimenter and subject were satisfied that the tasks could be performed as required. Some subjects did, however, move prematurely (i.e., while the goggles were still open) on one or a few NVF trials, so these were repeated at the end of the standard block.

### Data processing and definitions

Hand movement data were initially processed using custom-written programmes in Matlab (The MathWorks Ltd., Cambridge, UK) software. These generated separate ‘profiles’ of the reach velocity and spatial path (recorded from the wrist marker) and of the grip aperture (computed from the 3D distance between the thumb and finger markers) throughout each movement, along with a number of dependent measures of its kinematics. Definitions of several key landmarks in the movement were similar to those of previous studies (e.g., Melmoth and Grant 2006, 2012). The movement onset (MO) or ‘reaction’ time was defined as the period between initial lens opening (FV) or closure (NVF) and the moment when the wrist marker first exceeded a velocity of 50 mm/s in the forward (*y* axis) direction. Initial object contact at the end of the reach and its lifting at the end of the grasp were defined by the moments when the marker on the target was first displaced from its original position by > 1 mm and by > 10 mm, respectively, with the overall movement duration (MD) defined as the period between movement onset and object lifting. MO and MD times were used as general measures of the overall efficiency of the planning and execution phases, respectively.

To identify any specific movement sub-actions that retained advantages of binocular vision when it was available only at the planning stage, each subject’s performance under FV and NVF conditions was compared on a range of reach and grasp parameters. The reach was examined by six parameters, mainly derived from the wrist marker. Two were measures of its early dynamics—the peak velocity (PV) and the time to peak velocity (tPV); and one related to its end-stage—the duration of the low velocity phase (LVP) between peak deceleration and initial object contact. The others assessed the directness of the reach trajectory defined as the overall hand path length (HPL) between MO and object contact, and the presence of ‘errors’ during the final approach signified by pre-contact adjustments (i.e., extra re-accelerations/decelerations) in its velocity profile or as mis-reaches signified by extra forward and/or lateral deviations from a single, curved path in its spatial profile.

The grasp was examined by 12 parameters, mainly derived from the thumb and finger markers. Two were measures of its early phase—the peak grip aperture (PGA) at hand pre-shaping and the time to peak grip (tPG), with a further three assessing its end-stage dynamics—the grip closure time (GCT) between PGA and initial object contact; the period between this initial contact and the moment of minimum terminal reach velocity—a parameter termed reach–grasp coupling at object contact—and the grip application time (GAT) between initial contact and object lifting. Three assessed aspects of the end-point grasping accuracy—the grip size at initial object contact (GOC) and the presence of ‘errors’ signified by extra opening/closures in the thumb–finger aperture profile occurring during either the final approach to the target (pre-contact adjustments) or while manipulating it (post-contact adjustments) prior to the lift. Finally, four aspects of digit precision when forming the end-point grip were assessed, represented by the variability (standard deviations across trials) of the thumb and the finger marker positions at their moments of contact in both the horizontal/retinal image (*x*-axis) and depth/forward (*y*-axis) planes of the object.

### Statistical analyses

Because subjects completed relatively few trials of any given type, their movement kinematics were calculated from median values obtained by view. This was to better denote the central kinematic tendencies (Altman 1999) by minimizing analysis of several outlying data points arising from occasional atypical movements produced on the same, usually NVF, trial. The different types of adjustment/errors were expressed as the percent of trials on which they occurred, for which—to strike a balance—data from any such unusual movements were not censored. These data were analysed by repeated measures analysis of variance (ANOVA) in SPSS (SPSS UK Ltd, Woking) software. Separate ANOVA were first conducted on the overall data obtained across the three views in the FV and in the NVF conditions, to establish whether any general binocular advantages over monocular viewing with visual feedback available were also present when it was not. Selected parameters of interest were then entered into more detailed ANOVA which included the 2 object distances and 2 sizes as the within-subjects factors. Finally, Spearman’s rank correlation analyses were conducted on the overall data obtained for each view to identify any parameters that consistently co-varied under the FV and the NVF conditions, and so might be products of the same control mechanism(s). These analyses involved a large number of comparisons, so steps were taken to minimize reporting of Type 1 (false positive) errors. The Huynh–Feldt adjustment, which corrects degrees of freedom to offset data non-sphericity, was applied as needed in the ANOVA, with the Bonferroni correction for multiple pairwise comparisons used to identify the origin(s) of any main effects or interactions achieving the accepted significance level of *p* < 0.05. Significance was set at a more conservative threshold of *p* < 0.01 for the correlation analyses.

## Results

### Overall effects of view and feedback

Tables 1, 2, 3, 4 and 5 document the main effects of the 3 viewing and 2 feedback conditions, and any interactions between them, on the 20 parameters analysed collapsed across target distance and size. They include the outcomes of the separate ANOVA conducted by view within each feedback condition (see asterisks), as this: (1) provides confirmation that our subjects exhibited the typical range of normal binocular advantages reported in previous FV studies; and (2) helps identify those that were retained when binocular vision was only available for movement planning. Note that the (numerous) main effects of view were all due to a strong binocular FV advantage—irrespective of the NVF condition—because there were no significant differences at all between the subject’s dominant and non-dominant eye performance and because all main effects of feedback resulted from significant deteriorations in the NVF condition. Accordingly, the (fewer) feedback by view interactions achieving significance were driven by a selective loss of the normal binocular advantage when vision was only available for planning, as indicated by the greater %differences in binocular compared to monocular FV versus NVF performance.

**Table 1:** Average (median + sem) movement planning and execution times

Dependent measures	Condition	View			View (2,38)	Feedback (1,19)	Feedback × view (2,38)
		Bino	Dom eye	ND Eye	*p* values	*p* values	*p* values
Movement onset (ms)	Full vision No feedback (difference)	489 (17) 528 (18) (8%)	509 (17) 528 (21) (4%)	516 (17) 525 (20) (2%)	0.2 (NS)	0.2 (NS)	0.1 (NS)
Movement duration (ms)	Full vision*** No feedback* (difference)	854 (17) 1051 (69) (23%)	951 (33)^###^ 1098 (77) (15%)	979 (27)^###^ 1109 (70)^#^ (13%)	< 0.001	0.011	0.030

*Bino* binocular,* Dom* dominant,* ND* non-dominant,* NS* not significant

Asterisks represent main effects of view within each vision condition: *p < 0.05; ***p ≤ 0.001

Cross-hatches represent binocular advantages over the given monocular view: ^#^*p* < 0.05; ^###^*p* ≤ 0.01

(Difference) indicates the overall percent increases occurring in NVF compared to FV performance under each view

**Table 2:** Average (median + sem) reach and grasp dynamics/timings

Dependent measures	Condition	View			View (2, 38)	Feedback (1,19)	Feedback × view (2, 38)
		Bino	Dom Eye	ND Eye	*p* values	*p* values	*p* values
Reach parameters							
Peak velocity (mm/s)	Full vision*** No feedback** (difference)	746 (20) 710 (32) (– 5%)	711 (19)^##^ 686 (31)^#^ (– 4%)	700 (29)^##^ 682 (32)^#^ (– 3%)	< 0.001	0.2 (NS)	0.25 (NS)
Time to peak velocity (ms)	Full vision No feedback (difference)	272 (7) 278 (10) (2%)	271 (7) 276 (11) (2%)	277 (8) 280 (11) (1%)	0.4 (NS)	0.5 (NS)	0.9 (NS)
Low velocity phase (ms)	Full vision*** No feedback* (difference)	301 (12) 407 (34) (35%)	350 (17)^##^ 466 (45)^#^ (33%)	364 (18)^###^ 457 (44) (26%)	< 0.001	0.013	0.7 (NS)
Grasp parameters							
Time to peak grip (ms)	Full vision No feedback (difference)	499 (20) 534 (27) (7%)	512 (21) 532 (25) (4%)	517 (24) 550 (28) (6%)	0.08 (NS)	0.07 (NS)	0.8 (NS)
Grip closure time (ms)	Full vision*** No feedback** (difference)	236 (9) 301 (27) (28%)	286 (13)^###^ 360 (39)^#^ (26%)	298 (12)^###^ 358 (32)^##^ (20%)	< 0.001	0.042	0.4 (NS)
Grip application time (ms)	Full vision** No feedback (difference)	121 (5) 210 (21) (73%)	148 (8)^##^ 207 (20) (40%)	152 (8)^##^ 206 (20) (36%)	0.2 (NS)	0.001	0.039

All conventions as in Table 1, except main effects of view within each vision condition

**p ≤ 0.01; and binocular advantages over the given monocular view: ^##^*p* ≤ 0.01

**Table 3:** Average (median + sem) reaching accuracy and error rates

Dependent measures	Condition	View			View (2,38)	Feedback (1,19)	Feedback × view (2,38)
		Bino	Dom eye	ND eye	*p* values	*p* values	*p* values
Pre-contact velocity adjust (% trials)	Full vision** No feedback (difference)	5.1 (1) 14.6 (4) (186%)	15.6 (3)^##^ 22.9 (5) (47%)	16.8 (4)^#^ 18.2 (4) (8%)	0.017	0.1 (NS)	0.3 (NS)
Mis-reaches (% trials)	Full vision No feedback (difference)	2.8 (1) 4.0 (1) (43%)	5.6 (2) 9.1 (3) (62%)	4.2 (2) 7.3 (2) (74%)	0.014	0.2 (NS)	0.7 (NS)
Hand path length (mm)	Full vision** No feedback (difference)	294 (5) 298 (8) (1%)	308 (7)^##^ 307 (8) (0%)	306 (6) 307 (6) (0%)	0.004	0.8 (NS)	0.5 (NS)

All conventions as in Tables 1 and 2

**Table 4:** Average (median + sem) grasping accuracy and error rates

Dependent measures	Conditions	View			View (2,38)	Feedback (1,19)	Feedback × view (2,38)
		Bino	Dom eye	ND eye	*p* values	*p* values	*p* values
Peak grip aperture (mm)	Full vision*** No feedback (difference)	72 (2) 89 (3) (24%)	77 (2)^###^ 89 (3) (16%)	77 (2)^###^ 90 (4) (17%)	0.001	< 0.001	< 0.001
Grip size at contact (mm)	Full vision** No feedback (difference)	41 (1) 55 (2) (34%)	44 (1)^##^ 54 (2) (22%)	45 (1)^##^ 53 (2) (18%)	0.5 (NS)	< 0.001	0.02
Reach–grasp coupling at object contact (ms)	Full vision*** No feedback (difference)	29 (5) 126 (16) (334%)	50 (4)^###^ 109 (14) (118%)	55 (4)^###^ 121 (15) (120%)	0.2 (NS)	< 0.001	0.008
Pre-contact grip adjusts (% trials)	Full vision** No feedback (difference)	2.1 (1) 9.7 (2) (362%)	8.6 (2)^#^ 16.0 (5) (86%)	6.8 (2)^#^ 11.3 (3) (66%)	0.016	0.021	0.7 (NS)
Post-contact grip adjusts (% trials)	Full vision* No feedback (difference)	2.4 (1) 15.5 (3) (546%)	5.7 (1) 16.7 (3) (193%)	8.4 (2)^#^ 16.3 (3) (94%)	0.2 (NS)	< 0.001	0.4 (NS)

All conventions as in Tables 1 and 2

**Table 5:** Average (+ sem) initial digit contact variability

Dependent measures	Conditions		View			View (2,38)	Feedback (1,19)	Feedback × view (2,38)
			Bino	Dom eye	ND eye	*p* values	*p* values	*p* values
Thumb depth variability (mm)	Full vision*** No feedback (difference)		4.3 (0.4) 8.5 (0.7) (98%)	6.6 (0.4)^##^ 9.3 (0.7) (41%)	7.5 (0.7)^###^ 8.6 (0.5) (15%)	0.007	< 0.001	0.029
Thumb horizontal variability (mm)	Full vision No feedback (difference)		4.1 (0.4) 5.2 (0.5) (27%)	4.1 (0.4) 5.6 (0.6) (37%)	4.2 (0.4) 5.8 (0.4) (38%)	0.7 (NS)	< 0.001	0.8 (NS)
Finger depth variability (mm)	Full vision*** No feedback (difference)		3.2 (0.2) 6.1 (0.4) (91%)	5.3 (0.4)^###^ 5.8 (0.4) (9%)	5.2 (0.4)^###^ 5.8 (0.4) (12%)	0.011	0.001	0.002
Finger horizontal variability (mm)	Full vision* No feedback (difference)		3.9 (0.3) 6.6 (0.6) (69%)	4.9 (0.4) 7.7 (0.8) (57%)	5.2 (0.4)^#^ 7.9 (0.6) (52%)	0.024	< 0.001	1.0 (NS)

All conventions as in Tables 1 and 2

**Fig. 1:**
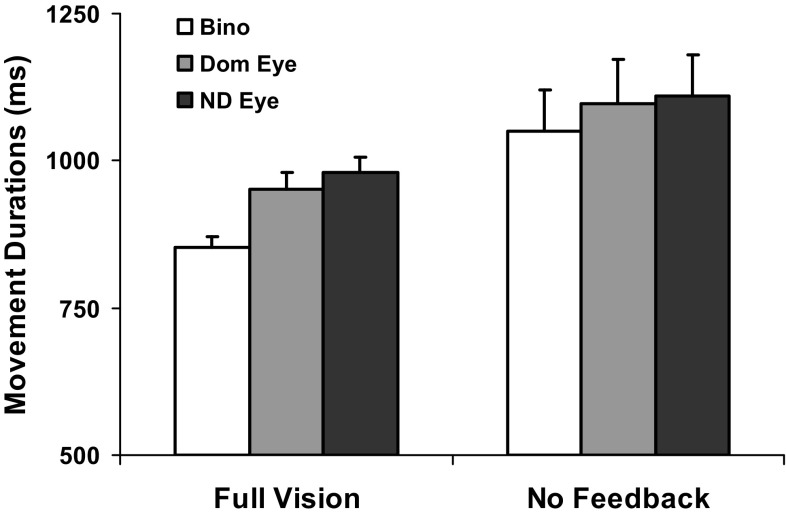
Average movement execution times under each viewing condition, with vision available throughout the movement (Full Vision) versus only during the planning stage (No Feedback). *Bino* binocular, *Dom* dominant, *ND* non-dominant. Error bars, SEM

Movement onset times (Table 1) were similar across views in both feedback conditions (all *p* > 0.1), with minor (millisecond level) extensions preceding NVF relative to FV trials unlikely to have caused any major decay to the stored representation of the action plan (Goodale et al. 1994; Westwood et al. 2005; Hesse and Franz 2010). There were, however, main effects of both view and feedback on movement execution times (Table 1; Fig. 1) which were generally faster with binocular compared to monocular viewing (*p* < 0.001) and when FV was available (*p* = 0.011). There was also an interaction between the two factors (*p* = 0.03). This was due to a marked reduction in the normal binocular advantage occurring in the absence of visual feedback, such that an apparent benefit was retained only with respect to the non-dominant eye (*p* = 0.045), with binocular movement durations increasing much more (by ~ 23%) than with the dominant and non-dominant eyes (by 13–15%) between the 2 feedback conditions.

**Fig. 2:**
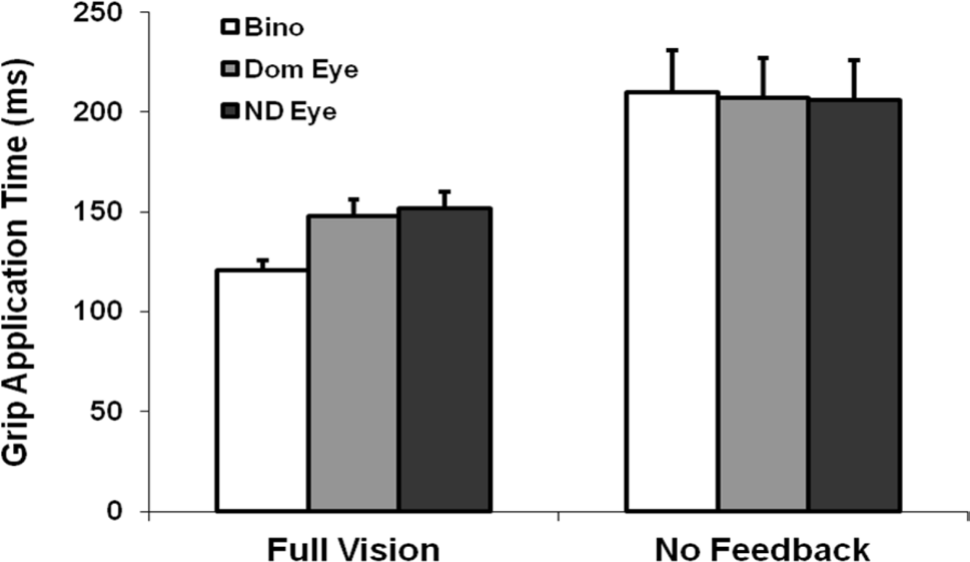
Average grip application times under each viewing and feedback condition. Other conventions, as in Fig. 1

The early timing parameters of the reach (tPV) and grasp (tPG) did not contribute to these effects on movement durations, since they were unaffected by view or by feedback (Table 2). By contrast, peak velocities of the reach, its LVP, and the grip closure and application times were all typically faster with binocular compared to monocular FV, with the three later timing measures markedly extended (by ≥ 20%) across views with NVF available. More surprisingly, somewhat faster binocular than dominant and/or non-dominant eye PVs, LVPs and GCTs were also observed in the absence of visual feedback. As a consequence—and contrary to our expectations—these three measures retained some benefit of binocular vision for planning (all *p* < 0.001), although grip application times did not. Instead, this was the only dynamic parameter to share a significant feedback × view interaction (*p* = 0.039) with MD. As shown in Fig. 2, this arose because GATs were essentially identical across views in the NVF condition due to much greater relative binocular (73%) compared to monocular (36–40%) increases between feedback conditions. Together these findings suggest that the selectively greater period of time spent between contacting and lifting the objects was mainly responsible for reducing the binocular advantage for overall movement execution times with NVF available.

**Fig. 3:**
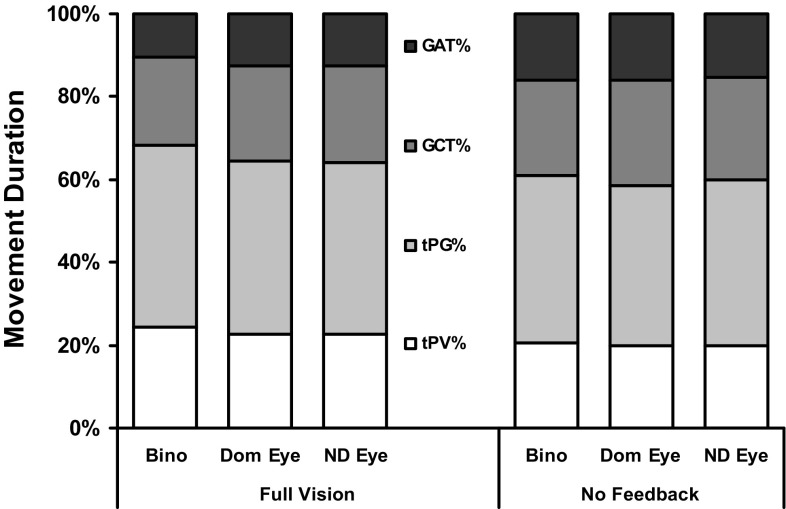
Average proportions of time spent in different phases of the reach-to-grasp as a function of overall movement durations by viewing and feedback condition. tPV%, tPG%, GCT%, GAT%; the percentages of time to peak velocity, time to peak grip aperture, the grip closure time, and grip application time, respectively. Other conventions, as in Fig. 1

To further examine this unexpected finding, (unplanned) analyses of the early (tPV, tPG), middle (LVP, GCT) and final (GAT) periods in the movement sequences, expressed as percentages of the movement durations, were undertaken by view and feedback. Supporting the above suggestion, these analyses showed (Fig. 3) that the relative times spent in the early reach (tPV%) and grasp (tPG%) phases were significantly reduced (by between 3 and 7%) in the NVF versus FV conditions (both *p* = 0.021), and especially (by 6%, on average), when binocular vision was available only for planning (feedback × view, both *p* < 0.005). But this was specifically because the GAT% was similarly increased under these same conditions (feedback, *p* = 0.002; feedback × view, *p* = 0.034), with no effects at all on the LVP% or GCT%.

Normal binocular viewing was associated with slightly shorter hand paths (although only with respect to the dominant eye, *p* < 0.05) and with markedly fewer pre-contact adjustments to the reach velocity than with monocular FV (Table 3). But because similar (non-significant) trends were present in the NVF condition, these measures of reaching accuracy (along with mis-reaches) were not significantly influenced by the absence of online visual feedback. By contrast, there were main effects of feedback on all of the accuracy/error measures of the grasp (Table 4). Participants formed wider grips at peak and at initial object contact accompanied by poorer (i.e., extended) reach–grasp coupling in the NVF condition, with the normal binocular advantages eliminated for these three measures (all *p* < 0.01) resulting in feedback × view interactions (all *p* ≤ 0.02) for each parameter. The loss of binocular advantage for the PGA was a surprise, because we expected this to benefit from the availability of disparity information when planning the grasp, whereas the adverse effects on the GOC and reach–grasp coupling were predicted due to the non-availability of online disparity cues during the hand–target approach. Subjects also adjusted their grip more often in both its pre- and post-contact phases with NVF available, especially on binocular compared to monocular NVF versus FV trials. As with pre-contact velocity corrections (Table 3), however, neither interaction achieved significance due to within- and between-subject variability in their rates of occurrence across the three views.

Both feedback and view, however, affected digit positioning at object contact (Table 5), with reduced precision (i.e., more trial-by-trial variability) in the initial placing of the thumb and the finger in both cardinal axes of the targets when online vision was not available to guide them (all *p* < 0.001) and when viewing monocularly (except for thumb contacts in the retinal image plane). Selective feedback × view interactions also occurred with respect to the variability in thumb and finger positioning in depth (both *p* ≤ 0.025), due to a much greater increases in binocular (91–98% differences) compared to monocular variability (of 9–41%) on NVF versus FV trials, which completely eliminated the normal binocular enhancement of grasping-in-depth precision.

### Effects of target object properties

**Fig. 4:**
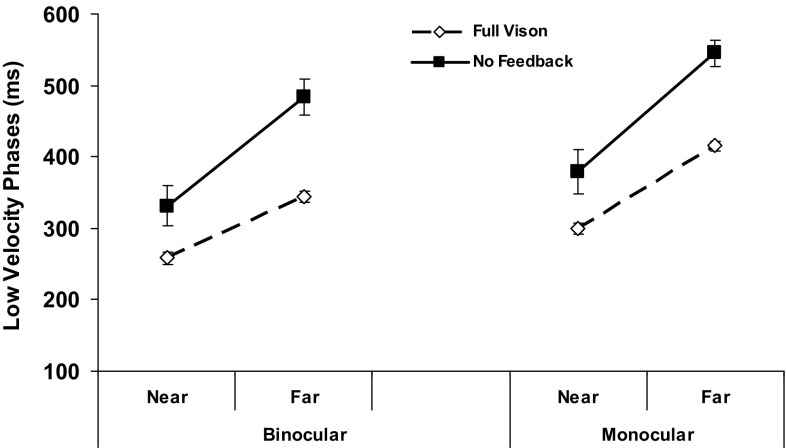
Average low velocity reach phases by object distance under binocular compared to monocular views, with vision available throughout the movement (Full Vision; open symbols, broken lines) versus only during the planning stage (No Feedback; filled symbols, solid lines). Monocular represents the mean of the dominant and non-dominant eye performances, between which there were no significant differences. Errors bars, SEMs. The LVP increased in the NVF compared to FV condition by ~ 60–80 ms for near but by ~ 130–140 ms for far targets across all views

Some advantages of binocular over monocular FV for prehension can be affected by the distance and/or the size/weight of the goal object. This section examines our hypotheses that, of the exemplars of these properties used in the present study, the farther target location and smaller/lighter of the two objects might pose particular challenges for movements conducted without visual feedback and so contribute to the loss of the normal binocular FV advantage occurring for the seven parameters concerned. Movement durations, the hand–target periods of the reach (LVP) and grasp (GCT) and the PGA all showed feedback × distance interactions (all *F*_(1,19)_ ≥ 5.1, *p* ≤ 0.035) due to significantly greater increases across all three views when subjects reached without vision to far compared to near targets. But there were no three-way distance-related effects on these or any other measure because, as exemplified by the LVP (Fig. 4), any relative increases occurring from near-to-far in the absence of feedback were similar for binocular and monocular movements. The target’s distance did not, therefore, selectively contribute to any loss of binocular advantage with NVF available.

**Fig. 5:**
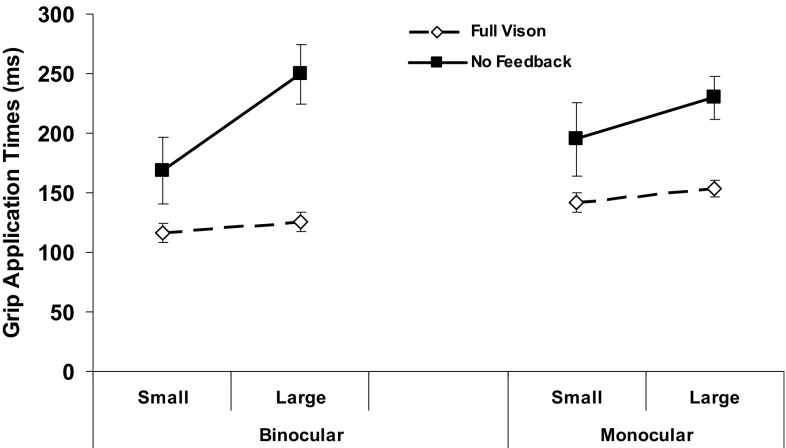
Average times spent applying the grip by object size. Other conventions, as in Fig. 4. The GAT increased in the NVF compared to FV condition by ~ 50–60 ms for the small object across all views, but by almost twice as much for the large object when binocular (~ 130 ms) versus monocular (~ 75 ms) vision was used to plan the grasp

Effects of object size were more complex. Movement durations, for example, were unaffected by this target property (*F*_(1,19)_ = 0.7, *p* = 0.4), because the subject’s tPG were slightly shorter for the small compared to larger object with a counterbalancing reverse difference for their GATs (both *p* ≤ 0.04). More importantly, grip application times showed a significant three-way (feedback × view × size) interaction (*F*_(2,38)_ = 3.4, *p* = 0.048). As shown in Fig. 5, this occurred because these times increased much more when subjects grasped the larger object in the absence of feedback, especially when using binocular vision to plan their grasp. A strong three-way trend (*F*_(2,38)_ = 3.0, *p* = 0.065) arising for the same reasons also occurred for reach–grasp coupling. In other words, extending earlier analyses (i.e., Figs. 2, 3), the increased time spent applying the grasp to, specifically, the larger/heavier object mainly accounted for the loss of the normal binocular advantage for these two temporal grasp parameters with NVF available.

**Fig. 6:**
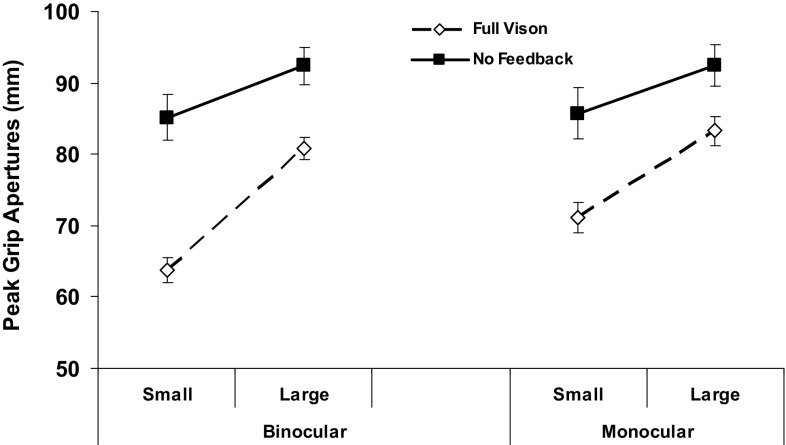
Average peak grip apertures formed during grasp preparation by object size. Other conventions, as in Fig. 4. The PGA increased in the NVF compared to FV condition by ~ 10 mm for the large object across all views, but more for the small object when binocular (~ 20 mm) versus monocular (~ 15 mm) vision was used to plan the grasp

**Fig. 7:**
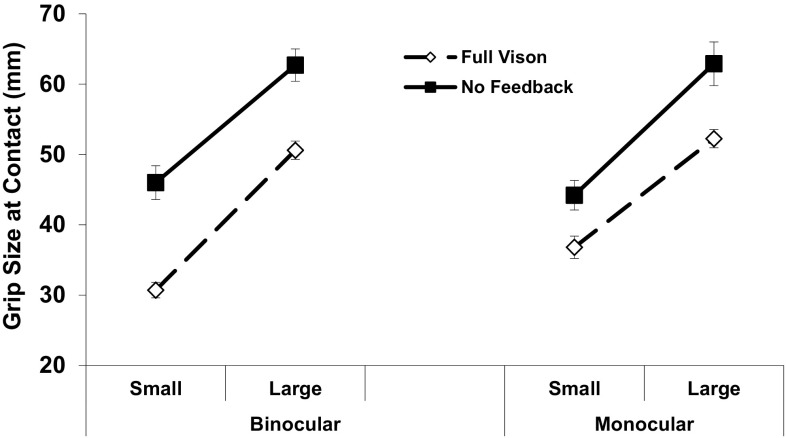
Average grip apertures at initial object contact by target size. Other conventions, as in Fig. 4. The GOC increased in the NVF compared to FV condition by ~ 12 mm for the large object across all views, but more for the small object when binocular (~ 16 mm) versus monocular (~ 8 mm) vision was used to plan the grasp

There were further opposing target size effects on spatial aspects of the grasp. The PGA and GOC both increased for the larger object, as would be expected, and so did the rate of pre-contact grip adjustments (all *p* ≤ 0.001) this being mainly driven by their much more frequent (~ 2.5-fold) occurrence when subjects were preparing to grasp the larger target with NVF available (feedback × size, *p* = 0.012). However, as hypothesized, the smaller object posed a particular challenge, with both the peak grip (Fig. 6) and grip size at contact (Fig. 7) increasing more for this compared to the larger target when grasping them in the absence of online vision. For the grip at contact, this overall effect was greater between binocular than monocular NVF and FV conditions (feedback × view × size, *F*_(2,38)_ = 4.4, *p* = 0.019), and there was a strong three-way trend (*F*_(2,38)_ = 2.5, *p* = 0.094) occurring for the same reason for the PGA. That is, increased grip sizes associated with the smaller/less stable object mainly accounted for elimination of the normal binocular advantage for these two measures of grasping accuracy with NVF available. The four measures of digit contact variability (Table 5) also increased more for the small *versus* large object (all *p* ≤ 0.001). However, unlike grip accuracy, there were no two- or three-way interactions related to the loss of the normal advantage for thumb or finger positioning-in-depth precision, suggesting that these were general deficits associated with removing online binocular feedback.

### Correlation analyses

**Fig. 8:**
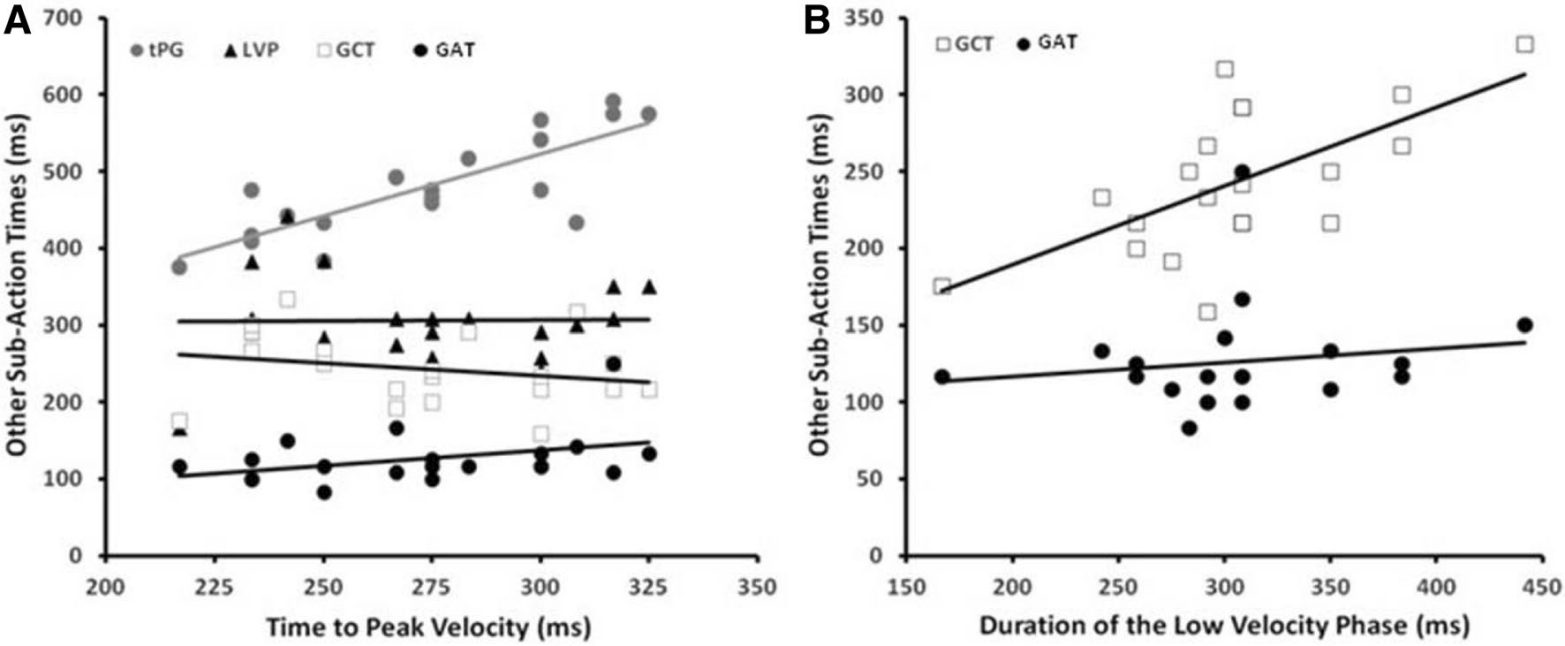
Correlations between the timing of different movement sub-actions performed under binocular full vision conditions across participants. **a** Their time to peak velocity *versus*; times to peak grip (tPG, grey circles; *R* = 0.75, p < 0.001); low velocity phase (LVP) durations (filled triangles; *R* = 0.18, *p* = 0.94); grip closure times (GCT, open squares; *R* = – 0.25, *p* = 0.30); and grip application times (GAT, filled circles; *R* = 0.34, p = 0.14). **b** Their LVP durations versus; GCT (*R* = 0.58, *p* = 0.007); and GAT (*R* = 0.27; *p* = 0.24)

With normal binocular and monocular FV, each sub-component of the movement timings was positively correlated in each participant with their own overall movement durations. As exemplified for binocular FV in Fig. 8, however, associations between the different sub-actions were highly selective. Specifically, the two early (tPV, tPG) and the two middle (LVP, GCT) components were positively correlated with each other under each view (*R* = 0.58–0.85, *p* ≤ 0.005), but there were no significant relationships between either of the early and the hand–target sub-actions or with the final grip application time (*R* = – 0.35–0.34, *p* > 0.15), which was positively correlated only with the efficiency of reach–grasp coupling at object contact (*R* ≥ 0.70, *p* ≤ 0.001). Correlations between tPV and tPG were usually similar across all trial types, whereas those between the LVP and GCT and for reach–grasp coupling and the GAT were generally stronger for the same object distance and/or size trial combination(s). The only other correlations were that grip adjustments during the final hand–target approach were associated (*R* ≤ – 0.50, *p* ≤ 0.01) with improved end-point grasping accuracy (smaller grip sizes at contact) and precision (less variable thumb and finger placements-in-depth), suggesting that they were mediated by online visual feedback. These findings are consistent with a tripartite sequence of selectively related sub-actions underlying normal reach-to-grasp movements.

**Fig. 9:**
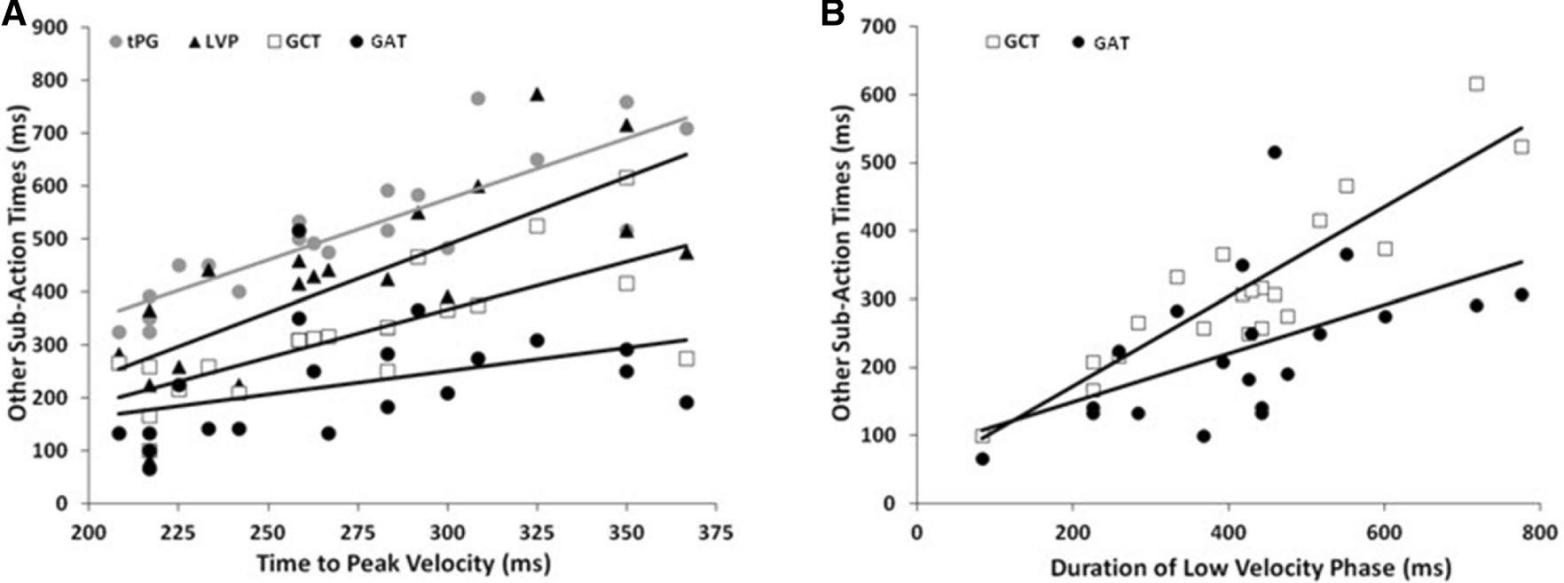
Correlations between the timing of different movement sub-actions performed under binocular no visual feedback conditions across participants. Conventions, as in Fig. 7. **a** Time to peak velocity versus; tPG (*R* = 0.84, p < 0.001); LVP durations (R = 0.71, *p* < 0.001); GCT (*R* = 0.77, *p* < 0.001); and GAT (*R* = 0.57, p = 0.010). **b** LVP durations versus; and GCT (*R* = 0.82, *p* < 0.001); and GAT (*R* = 0.62, *p* = 0.003)

All of these selective relationships were absent or eroded, however, whenever vision was only available for movement planning. As exemplified for binocular NVF in Fig. 9, movement durations and all of its sub-actions (now including reach–grasp coupling) were positively correlated with each other across participants and most trial types. Moreover, subjects who produced shorter duration movements consistently reached faster (i.e., with higher PV; *R* ≤ – 0.50, *p* ≤ 0.01) usually accompanied by wider (safer) peak grips at hand pre-shaping (*R* ≥ 0.66, *p* ≤ 0.001). Also unlike normal viewing, there were no associations at all between any aspect of pre-contact and end-point grasping performance.

## Discussion

The present study replicated evidence that reach-to-grasp performance with full binocular vision is faster (e.g., increased PV; shorter MD, LVP, GCT, GAT), more accurate (e.g., better grip-to-object size scaling at peak and contact; fewer errors and corrections) and precise (e.g., less variability in initial digit positioning in the object’s depth plane) than when viewing is restricted to one eye. It also replicated evidence that performance is generally slower—with significantly altered sub-action timing patterns—less accurate and imprecise when vision is available only for planning the up-coming actions. Only the earliest movement parameters (PV, tPV, tPG) and the directness of the reach path (HPL, mis-reaches) were hardly affected at all by the NVF condition and thus seemingly under exclusive feedforward control. These results confirm that our current subjects exhibited the typical binocular advantages and use of visual feedback for most aspects of prehension performance typically reported for normal adults.

Against these important pre-conditions, there were four main findings related to the major study aims. First, two key features of the reach, its PV and LVP duration, retained reduced, but significant, advantages from binocular viewing for planning hand transport, whereas those associated with virtually every aspect of the grasp were eliminated when binocular vision was absent after movement onset. The two exceptions to this were the tPG and GCT (Table 4) which temporally overlap the tPV and LVP, respectively, and were correlated with these transport components (Figs. 8, 9). Second, the losses of binocular advantage were unrelated to the distance of the goal object, whereas its size sometimes mattered. In particular, the larger (and heavier) object was associated with elimination of the normal binocular benefits for efficient reach–grasp coupling and grip application, with the smaller (less stable) object linked to the loss of normal binocular grasping accuracy at grip pre-shaping and initial object contact. Third, prolonged grip application times were responsible for altering the overall movement pattern in the NVF condition (Fig. 3) and for eliminating the normal binocular advantage for faster movement times. Fourth, durations of the early, middle/pre-contact and final/post-contact movement periods were uncorrelated with FV available, supporting the notion that there are differences in their modes of control. But they were all correlated with each other with NVF available, consistent with being outcomes of a single feedforward mechanism derived from the stored memorial representation of the up-coming tasks generated during the 1-s previews.

### Some binocular advantages for planning hand transport

The first finding suggests that additional sources of binocular information available during the task previews were sufficient to enhance dynamic aspects of the reach. A general (main) effect of binocular viewing was also revealed by some improvements its spatial aspects (i.e., fewer mis-reaches, shorter hand paths; Table 3), implying a further advantage over monocular vision for programming the hand trajectory. One potential source of these binocular planning advantages could be ocular vergence-derived cues to the target’s absolute egocentric distance, a key determinant of the transport kinematics. Saccade and vergence latencies following target presentation are reported to be around 200 ms (Yang et al. 2002) and so are short enough that our subjects should have had ample opportunity to bi-fixate the goal objects in the 1 s planning time they were allotted. Moreover, vergence-related distance information has been shown to systematically influence the PV, LVP duration and amplitude of binocularly programmed reaches in normal adults (Mon-Williams and Dijkerman 1999; Melmoth et al. 2007); that is, some of the very measures of reaching performance for which our subjects exhibited some binocular planning advantages. This is consistent with our second finding that differences in the target’s distance, while affecting several reach and grasp parameters, was not a factor in eliminating any of our subjects’ normal binocular advantages in the NVF condition.

It was, nonetheless, surprising that any residual binocular advantage for reducing the duration of the final reach LVP (and the co-varying GCT) was observed when NVF was available, since these normal benefits have been widely attributed (e.g., Watt and Bradshaw 2000; Loftus et al. 2004; Melmoth et al. 2007, 2009; Anderson and Bingham 2010) to online processing (e.g., ‘nulling’) of horizontal disparities signifying the receding space between the hand/digit-tips and the goal object. One possibility is that vergence-specified target distance estimates—possibly with contributions from vertical image size disparities in the two eyes (Rogers and Bradshaw 1993)—were reliable enough to partly override the loss of online disparity cues normally used in the final approach. This would accord with the idea that data required for reach programming need only to be accurate enough to aid hand transport to the target while braking early enough to avoid colliding heavily with it (e.g., Loftus et al. 2004; Melmoth and Grant 2006). It would also align with evidence that people who cannot process horizontal disparities, because they lack stereovision, seem to adopt a strategic trade-off in which they dispense with spending time estimating hand–target depth relations during the LVP and GCT in favour of using non-visual, haptic feedback to correct their grip when subsequently contacting the goal object (Melmoth et al. 2009).

Indeed, binocular NVF compared to FV trials were associated with an especially marked (~ threefold) increase in corrections to the reach velocity during the braking period, so that the normal advantage of binocular over monocular vision for reducing the need for these was lost in the absence of online vision (Table 3). One interpretation of these adjustments is that they represent a strategic undershooting of the target, deliberately produced for safety reasons to prevent the programmed reaches colliding hard with the unseen targets. Another relates to observations by Wolpert et al. (1995) that when subjects reach in the dark in the absence of a target, they slightly—but consistently—over-estimate the distance that their hand has actually travelled indicating a systematic bias in predictive reach control. Since it is likely that subjects will do this in the presence of a target too, this would require them to generate an extra acceleration/deceleration in their end-phase reach so as to make contact with it. Either way, the similar frequency of these corrections across views only in the NVF condition represents one of the few indicators in our data of an equivalence between binocular and monocular reach planning.

### Little or no binocular advantage for planning the grasp

By contrast, there were multiple equivalences between using binocular or monocular vision for grasp planning in the NVF condition, supporting previous reports that the normal binocular advantage for enhancing most aspects of grip timing, accuracy and precision derive from online disparity processing. Importantly, our data now indicate that this may apply to formation of the PGA at hand pre-shaping, contrary to the common assumption (e.g., Melmoth and Grant 2006) that the normal advantage for this grasp parameter arises from exploiting additional disparity cues to the target’s solid 3D properties at the programming stage. Yet the width of the PGA was nearly identical when our subjects formed their grasp for both small and large targets regardless of whether this extra information was present during the task preview. Instead, it was only when binocular vision was available online that an advantage for improved PGA sizing occurred, selectively related to the smaller of the two objects (Fig. 6). This latter observation is not unusual, as we (Melmoth and Grant 2006) and others (e.g., Servos et al. 1992; Watt and Bradshaw 2000; Keefe and Watt 2009; Keefe et al. 2011) have previously found that monocular viewing is associated with a relative PGA ‘over-sizing’ for smaller (e.g., ≤ 40 mm wide) targets. The effect is typically ascribed to uncertainty in judging an object’s true size when planning monocular grasps, with selective over-sizing for smaller/less stable targets a precautionary strategy designed to ensure their successful capture without knocking them over. But our data suggest that the addition of disparity cues to target solidity during grip planning does little to improve confidence in these judgements.

This would be consistent with evidence, some of which we previously overlooked, that early online vision of the target is critical for PGA formation. More specifically, it has been shown that abruptly increasing the apparent size of an object at the moment of movement onset after subjects have planned their grasp for a smaller target results in gradual widening of the evolving grip to re-scale the PGA to the new target’s dimensions (Paulignan et al. 1997; Karok and Newport 2010), with wider/safer PGAs also gradually produced when vision is suddenly occluded during the earliest (acceleration) phase of the reach (Fukui and Inui 2006, 2013). Chen and Saunders (2016) have further shown that changing the size of the object-to-be-grasped by introducing a brief mask just after the peak reach velocity results in accurate corrections to the grip at contact appropriate for the dimensions of the new target. One possibility is that online disparity processing early in the movement is involved in comparing the evolving grip aperture with the target’s dimensions to improve PGA scaling, whereas afterwards it is more involved in comparing relative 3D positions of the digit tips and their pre-selected contact points on the object in the hand–target approach period, when our correlation analyses showed that adjustments to the closing aperture can enhance end-point grip accuracy and precision.

Both this specific conclusion regarding the PGA and our more general one regarding the very limited role of binocular vision in grasp planning, however, require some qualification. First, although the data shown (Fig. 6) support that conclusion and are similar to those obtained by Watt and Bradshaw (2003), we should note that the relevant three-way interaction did not quite meet the criterion of statistical significance. Second, Keefe et al. (2011) previously found that better PGA scaling for smaller targets was reduced under binocular compared to monocular NVF conditions, although not as markedly as we did. Their study involved targets defined only by stereo/disparity- or by texture/perspective-cues in a virtual reality set-up, with observers allowed to grasp real, presentation-matched, objects at the end of the movements to provide veridical haptic feedback. But it could be that their subjects inevitably placed a greater weighting on the disparity information present within the limited subset of available cues during binocular grasp planning than did ours, who were operating in a more natural environment, richer in alternative sources of monocular 3D information. We found an overall correlation between shorter movement times and wider PGAs in this condition. This relationship suggests a speed–accuracy trade-off (e.g., Wing et al. 1986; McIntosh et al. 2018), whereby the faster-moving participants—perhaps in an effort to grasp the more challenging object before their memorial representation of it had substantially degraded—may have built an extra safety margin into their PGA which contributed to the more marked effect we observed. Consistent with this possibility, post hoc analyses revealed a significant correlation between shorter movement durations and wider peak grips when our subjects binocularly planned to grasp the smaller (Spearman’s $\rho$ = 0.54, *p* = 0.014), but not the larger ($\rho$ = 0.09, *p* = 0.7), object in the absence of visual feedback. We do acknowledge, though, that in other studies more comparable to ours (e.g., Jakobson and Goodale 1991; Whitwell et al. 2008; Keefe and Watt 2009; Hesse and Franz 2010) binocular PGA scaling was not so affected under NVF conditions.

In this context, we only used the same two objects as targets which the subjects picked up at the end of their movements. This may have provided them with familiarity-based information derived from haptic feedback and from retinal image size cues which have sometimes (Marotta and Goodale 2001; Keefe and Watt 2009)—although not always (McIntosh and Lashley 2008; Borchers et al. 2011)—been suggested to be more beneficial for calibrating monocular compared to binocular grasps. The NVF trial blocks also always followed the FV blocks providing further opportunities for short-term associative learning of the specific object presentations to influence performance in the absence of online vision. An important new finding in these regards was that the altered overall movement patterns occurring in the NVF condition across all three views (Fig. 2) resulted mainly from a longer proportion of time spent in contact with the objects during their manipulation. The relevant dependent measure, the grip application time, corresponds to the period during which the thumb and finger secure the target and generate the grip and load forces needed to lift it. This period is known to increase with target weight (Weir et al. 1991) and is considered to be under predictive control as such learned representations of an object’s material properties are reported to play an increasing role in planning the scaling of these forces in advance of repetitive lifts (Johansson and Westling 1988) with purely visual analyses of the object’s likely size–weight relationship correspondingly subordinated as it becomes more familiar (Mon-Williams and Murray 2000). The fact that our subject’s grip application times increased across all views in the NVF trial blocks is, therefore, opposite to the effect expected of a strong contribution of familiarity-based object knowledge in planning its lift. However, we also found that the selective advantage of binocular vision for reducing the time spent in contact with the larger/heavier object was completely lost when it was not available to guide the grasp (Fig. 5). This effect occurred, at least in part, because the relative increase in the GAT for this object was much smaller in the monocular (~ 75 ms) compared to binocular (~ 130 ms) NVF conditions, which is in line with the possibility that familiarity may have been more useful for grasp planning with one eye.

As in Weir et al. (1991) and in our previous work (Melmoth and Grant 2006), we observed two main types of object contact ‘error’ in the grip profiles obtained from our current subjects. One involved no change at all in the size of the grip aperture once contact had been established, but with an unusually long time spent before executing the lift. This indicates that although their digits were initially well placed on the object, subjects appeared to require confirmation of the grip’s stability via haptic feedback before picking it up. In fact, this type of accurate, but prolonged, grip application occurred less commonly across all views in the NVF compared to FV conditions (not shown). The other involved a corrective re-opening and closing of the digits, indicating that their initial contact with the object was inaccurate and that haptic information was being used in a feedforward–feedback fashion to shift them into more secure positions. The occurrence of this type of post-contact grip adjustment increased significantly in the NVF condition, and most markedly after planning the grasp binocularly (Table 4). These observations extend our arguments above by suggesting that the loss of binocular advantage for the GAT was due to inaccuracies and inconsistencies in end-point thumb and/or finger contacts with the objects, particularly in their depth plane (Table 5), with resolution of these difficulties mediated by greater dependence on haptic digit–object interactions during the contact period. Melmoth et al. (2009) found that adults with selectively reduced or negative disparity processing capabilities exhibit a similar set of deficits in end-point grasping accuracy and precision—including prolonged grip application times on heavier objects and frequent post-contact grip adjustments—with closed-loop binocular viewing. As implied above, the non-availability of online stereo/disparity information, therefore, most likely accounted for the pattern of end-point binocular grasping deficits in the current NVF condition, even though the timing of the grip aperture closure period was less affected.

We conclude that binocular viewing during prehension planning is associated with some slight improvements, over monocular vision, in the feedforward/predictive programming of faster velocity (c.f., Jackson et al. 1997) and straighter reaches with faster hand–target approach times, whereas it provides no obvious benefits for grasping, including PGA formation. Such dissociations, even if contrary to our original thinking, are to be expected since proficient performance of different phases of the transport and grip components of prehension are generally considered to depend on analysis of different types of visual information by anatomically and functionally distinct superior parietal–dorsal premotor (dorsomedial) and intraparietal–ventral premotor (dorsolateral) cortical networks, respectively (Rizzolatti and Matelli 2003; Grafton 2010). Perhaps not coincidentally, given our findings, one of the dorsomedial network areas of superior parieto-occipital cortex (SPOC) appears to be primarily—if not, exclusively—concerned with the automatic encoding of target information needed for planning the reach (Pisella et al. 2000; Gallivan et al. 2009; Lindner et al. 2010; Vesia et al. 2010; Glover et al. 2012) and is a selective processing site of near-space vergence-derived signals (Quinlan and Culham 2007).

It is less clear whether any areas in the dorsolateral grasp circuit are selectively involved in only its pre-movement planning (Glover et al. 2012). This would include the anterior intraparietal (AIP) area, known for some time to be necessary for both deciding on and preshaping the optimal grip for different types of graspable objects (Gallese et al. 1994; Binkofski et al. 1998; Murata et al. 2000; Begliomini et al. 2007) and to be active when subjects precision grasp 3D objects in the absence of online vision (Culham et al. 2003). In fact, evidence from various sources (Toni et al. 2007; Grafton 2010; Verhagen et al. 2008, 2012; Begliomini et al. 2014) suggests that AIP can rapidly formulate grasp plans weighted to meet the spatial accuracy demands of the task and based on integrating whatever monocular pictorial and/or binocular disparity cues seem to be most informative about the target object along with any prior knowledge of its properties. But it then quickly switches roles to dynamically control the grasp online to ensure that the ultimate action goal is successfully achieved. Our present and other data (e.g., Servos et al. 1992; Bradshaw and Elliot 2003; Loftus et al. 2004; Melmoth and Grant 2006; Lee et al. 2008; Anderson and Bingham 2010) converge on the conclusion that it is in this latter role that binocular vision usually makes its most significant contributions to the proficiency of prehension movements.

The preceding arguments have followed those of our original work (Melmoth and Grant 2006) in adhering to a commonly accepted conceptualization of prehension as requiring multi-factorial control of near-sequential reach–grasp–manipulate components (Jeannerod 1984), for which we have provided some support (Fig. 8). An alternative framework suggests that it more simply involves independent control of the thumb and index finger in aiming and guiding them to opposing contact points on the goal-object (Smeets and Brenner 1999). Evidence shows that, usually, either the thumb or the finger leads the way to make an initial soft landing at its pre-selected site on the target (Haggard and Wing 1997; Mon-Williams and McIntosh 2000; Melmoth and Grant 2012; Cavina-Pratesi and Hesse 2013; Grant 2015; Voudouris et al. 2016, 2018), the programming of which would likely benefit from binocular vergence cues to the absolute distance that the given digit needs to travel, as they do for single finger aiming-in-depth (Melmoth et al. 2007). The framework further suggests that the PGA is merely an emergent property of each digit’s independent trajectories, rather than a specifically controlled grasp parameter, with the thumb–finger separation scaling for target size because the movement of each digit needs to incorporate a margin for clearing the object’s opposing sides. Online binocular disparity processing could provide advantages for ensuring such clearances occur so that unintended collisions with object are avoided and that the digits then approach their contact sites along the pre-selected opposition axis through the target, as the framework specifically contends (Smeets and Brenner 1999; Verheij et al. 2014). These re-formulations of our conclusions are important, because they indicate that our main findings are compatible with key precepts of this alternative model.
